# The role of postoperative antibiotics in preventing surgical site infections in uncomplicated appendicitis

**DOI:** 10.1016/j.amsu.2021.01.037

**Published:** 2021-01-19

**Authors:** Sabry Abounozha, Rashid Ibrahim, Faisal Mohammed Alshehri, Hossam Nawara, Awad Alawad

**Affiliations:** a Northumbria Healthcare NHS Foundation Trust, Northumbria, UK; b University Hospitals Plymouth NHS Trust, Plymouth, UK; c Imam Muhammad Ibn Saud University Hospital, Riyadh, Saudi Arabia; d Wales University Hospital, Cardiff, UK

**Keywords:** Uncomplicated appendicitis, Appendicectomy, Appendectomy, Postoperative, Antibiotic, Surgical site infections, Intra-abdominal collection

## Abstract

A best evidence topic has been constructed using a described protocol. The three-part question addressed was: In patients who underwent appendicectomy for uncomplicated appendicitis is the use of postoperative antibiotics associated with lower rates of surgical site infections?

The search has been devised and 6 studies were deemed to be suitable to answer the question. The outcome assessed was the efficiency of postoperative antibiotic therapy in decreasing the rate of surgical site infections in uncomplicated appendicitis. Authors recommend against the use of postoperative antibiotics based on the supported evidence. Hence, its usage was not associated with lower rates of surgical site infections. On the contrary, it might increase the cost, postoperative morbidity and length of stay.

## 1 Introduction

This BET was constructed using a framework outlined by the International Journal of Surgery [1]. A BET provides evidence-based answers to common clinical questions, using a systematic approach of reviewing the literature.

## 2 Clinical scenario

After performing an appendicectomy for uncomplicated or simple appendicitis for one of your patients. You wonder whether the addition of further postoperative antibiotic therapy would decrease the chances of developing superficial or deep surgical site infections?

## 3 Three-part question

In [patients who underwent appendicectomy for uncomplicated appendicitis] is [the use of postoperative antibiotic] associated with lower rates of [superficial or deep surgical site infections]?

## 4 Search strategy

The search was conducted as following:

Embase 1974 to 2020 and MEDLINE® 1946 to November 2020 using the OVID interface. The results were limited to English articles and human studies:

[simple appendicitis OR uncomplicated appendicitis] AND [appendicectomy OR appendectomy] AND [postoperative antibiotic OR post-operative antibiotic] AND [surgical site infection OR SSI OR intra-abdominal collection OR intraperitoneal collection OR pelvic collection].

## 5 Search outcome

6963 articles were found. Out of these 9 deemed to be suitable and met the criteria of our search. 6954 articles were excluded as they included duplicate, conference abstracts and irrelevant articles based on titles and abstracts. 9 full-text articles were screened and assessed for eligibility. 6 out of 9 articles were chosen as they provided the best evidence and were confined to postoperative antibiotics administration in simple or uncomplicated appendicitis and their role to decrease the postoperative surgical site infections which is the core of our topic. The full articles reviewed carefully to ensure including the best evidence to answer the question.

Different papers have defined acute simple/uncomplicated appendicitis differently but all of them have agreed to define it as inflamed nonperforated appendicitis. Surgical site infections (SSIs) defined as both superficial and deep surgical site infections which occur within 30 days postoperatively. Superficial surgical site infections were defined as superficial wound infection which requires antibiotic therapy or laying the wound open along with wound care. Deep surgical site infections defined as the fluid collection inside the peritoneal cavity confirmed by ultrasound or computed tomography that requires drainage or antibiotic therapy.

## 6 Result

(Please refer to the table).

**Table:** 

Author, date of publication, journal and country	Study type and level of evidence	Patient group	Outcomes	Follow up	Key results	Additional comments
Le et al. [2], 2009, The American Journal of Surgery, USA	Retrospective cohort study, level III	The study included 763 patients who underwent appendicectomy for nonperforated appendicitis over a 10-year period from 1997 to 2007, 2 groups: 1-who received postop antibiotic and 2-who did not	Evaluation of postoperative development of surgical site infections (SSIs) and compare the incidence rates between the two groups	Both groups had a median follow-up of 14 days	The use of postoperative antibiotics in patients with nonperforated appendicitis does not decrease the rate of SSIs, while it may increase the cost of care. No significant differences in the rates of all SSIs (10% vs 9%, P = 0.64), superficial SSIs (9.3% vs 5.4%, P = 0.13), deep SSIs (0.3% vs 0.5%, P = 1.0), organ space SSIs (2.8% vs 2.7%, P = 0.87)	Large sample size, Single centre, included patients with intraoperative finding of normal appendix, minor antibiotics regimen variability, selection bias cannot be excluded, risk of documentation error, 35% of patients surgically treated for nonperforated appendicitis during the study period was excluded because they were lost to follow-up or because of an absence of follow-up documentation
Coakley et al. [3], 2011, the American College of Surgeons journal, USA	Retrospective cohort study, level III	The study included 728 cases; 334 of these patients received postoperative antibiotics and 394 did not	Comparing the incidence of postoperative surgical site infection after appendectomy in patients with Non-perforated appendicitis between the two groups	Median follow-up: Antibiotics group = 14.8 days No antibiotics group = 14.5 days	Postoperative antibiotic treatment for nonperforated appendicitis did not reduce infectious complications and prolonged length of stay while increasing postoperative morbidity	Large sample size, confounding factors were noted and compared the two groups accordingly, univariate and multivariate analysis single centre, there was a difference between antibiotic regimen in some cases, the group which received post-operative antibiotic did contain a higher percentage of patients with gangrenous disease
Hussain et al. [4], 2012, Journal of the College of Physicians and Surgeons Pakistan, KSA	Randomized controlled trial, level II	The study included 377 patients were admitted with the clinical diagnosis of acute appendicitis, 195 patients received only a single dose of pre-operative antibiotics (group A); while 182 patients received additional one dose of postoperative antibiotics (group B)	Comparing the incidence of postoperative surgical site infection after appendectomy in patients with Non-perforated appendicitis between the two groups	Both groups were followed-up for 30 days	Statistically there was no significant difference in rates of SSIs between both the groups (p = 0.9182)	Single centre, randomized, power calculation was not mentioned, study is not blinded, diabetic, immunocompromised and pregnant patients were excluded, risk of bias, patients lost in follow up were not included, the study was designed for open appendicectomy only
Hughes et al. [5], 2013, Surgical Infections Journal, UK	Prospective cohort study, level III	The study included 188 cases who had simple appendicitis and 78 for complicated appendicitis. In simple/uncomplicated appendicitis 104 patients did not receive post-operative antibiotics and 84 who did	The main outcome measure was the development of an Intra-abdominal infection between the two groups	All patients were followed-up for 30 days postoperatively	Authors have concluded that in simple appendicitis, post-operative antibiotics may not be beneficial at all. No significant difference in developing intra-abdominal infection between the two groups (P = 0.63)	Single centre, small sample size, selection bias cannot be excluded, non-standardized antibiotic regimen
Rafiq et al. [6], 2015, Journal of Pakistan Medical Association, Pakistan	Randomized controlled trial, level II	The study included 390 patients, 192 (49.2%) were in Group A (received a single dose of cefuroxime sodium and metronidazole half-an-hour before induction) and 198 (50.7%) in Group B (received one more dose of the same antibiotics postoperatively)	To evaluate the role of postoperative antibiotics in reducing surgical site infections after appendectomy for non-perforated appendicitis	Both groups were followed-up for 6 weeks	A single pre-operative dose of antibiotic regimen had the same efficacy in preventing surgical site infections as when the same regimen was repeated postoperatively. Number of surgical site infections were 15 (7.8%) in Group A and 18 (9.1%) in Group B (p = 0.65)	Randomized, Single Centre, Uniform guidelines of antibiotic protocol, did not mention the randomization and blinding process, no power calculation, patients with immunocompromised status, diabetes, pregnancy, body mass index above 25 were excluded, bias cannot be excluded, study designed for open appendicectomies only, patients who were lost in follow up were not included
Moosavi et al. [7], 2017, Caspian Journal of Internal Medicine, Iran	Randomized controlled trial, level II	The study included 152 patients who underwent appendicectomy for nonperforated appendicitis. Randomized into two groups, Group A patients received a single dose of preoperative antibiotics (ceftriaxone and metronidazole) and group B patients received the same regimen, in addition, antibiotics were administered 24 h postoperatively	To determine the role of postoperative antibiotics in reducing surgical site infections (SSIs) and abscess formation after open appendectomy	Both groups were followed-up for 30 days	Single dose of preoperative antibiotics was sufficient in reducing SSIs after appendicectomy. Postoperative antibiotics did not add an appreciable clinical benefit in these patients. The incidence rate of surgical site infections was statistically insignificant between the two groups	Randomized, single centre, Uniform guidelines of antibiotic protocol, did not mention the randomization and blinding process, no power calculation, patients with immunocompromised status, diabetes, heart failure, anaemia, pregnancy were excluded, bias cannot be excluded, study designed for open appendicectomies only

## 7 Discussion

Post appendicectomy SSIs is one of the common adverse events affecting patient safety worldwide [8]. Preoperative antibiotic therapy is a well-established and evidence-based practice which was found to be efficient to decrease the incidence of postoperative SSIs [9]. However, up to date there is no clear consensus about the role of post appendicectomy antibiotic therapy in patients with simple or uncomplicated appendicitis. Therefore, this search was conducted.

In 2009, Le et al. [2] conducted a retrospective cohort study which included 763 patients who underwent appendicectomy for nonperforated appendicitis over a 10-year period. They have classified the patients into 2 groups: the first group received postoperative antibiotics and the second did not. The authors found that there are no significant differences in the rates of all SSIs (10% vs 9%, P = 0.64) and they have concluded that the use of postoperative antibiotics in patients with nonperforated appendicitis does not decrease the rate of SSIs, while it may increase the cost of care.

In 2011, Coakley et al. [3] came up with the same conclusion and added that postoperative antibiotic therapy can increase the length of stay and the postoperative morbidity. His cohort retrospective study included 728 patients.

Hussain et al. [4] in 2012, conducted a randomized controlled trial which included 377 patients. 195 patients received only a single dose of preoperative antibiotics. While182 patients received an additional one dose of postoperative antibiotics. They concluded that there is no significant difference in rates of SSIs between both the groups (p = 0.9182).

In 2013, Hughes et al. [5] devised a retrospective cohort trial. The study included 188 cases who had simple appendicitis and 78 for complicated appendicitis. In simple/uncomplicated appendicitis 104 patients did not receive postoperative antibiotics and 84 who did. Authors found that there is no significant difference in developing intra-abdominal infection between the two groups (P = 0.63) and concluded that in simple appendicitis, postoperative antibiotics may not be beneficial at all.

In 2015 and 2017, both Rafiq et al. [6] and Moosavi et al. [7] conducted two randomized controlled trials which included 390 and 152 patients consecutively. Both studies concluded that Single dose of preoperative antibiotics was sufficient in reducing SSIs after appendicectomy.

All the above-mentioned studies have strongly agreed on the efficiency of preoperative antibiotic usage for decreasing the incidence of SSIs and that postoperative antibiotics were not beneficial in case of uncomplicated appendicitis.

The observed limitations are the possibility of selection bias and the variability of the selected antibiotic regimen in some of the studies. However overall, they came up with unified strong recommendations.

## 8 Clinical bottom line

Six studies including three randomized controlled trials have strongly recommended against the administration of postoperative antibiotics in case of uncomplicated or simple appendicitis as none of the studies has shown evidence of lower SSIs rate with its use. On the contrary, it might increase the cost, length of stay and postoperative morbidity.

## Ethical approval

Not applicable.

## Sources of funding

None.

## Author contribution

SA: devised the idea of the study, conducted literature search and wrote the paper.

RI: assisted in literature search editing and writing the paper.

FA: assisted in literature search and collecting the data.

HN: assisted in literature search and writing the paper.

AA: assisted in writing and editing the paper.

## Consent

Not Applicable.

## Registration of research studies

Name of the registry: not applicable
Unique Identifying number or registration ID
Hyperlink to your specific registration (must be publicly accessible and will be checked)

## Guarantor

Sabry Abounozha (SA), Rashid Ibrahim (RI), Faisal Mohammed Alshehri (FA), Hossam Nawara (HN), Awad Alawad (AA).

## Declaration of competing interest

None.
